# DNA-binding protein prediction using plant specific support vector machines: validation and application of a new genome annotation tool

**DOI:** 10.1093/nar/gkv805

**Published:** 2015-08-24

**Authors:** Graham B. Motion, Andrew J. M. Howden, Edgar Huitema, Susan Jones

**Affiliations:** 1 Division of Plant Sciences, University of Dundee at the James Hutton Institute, Invergowrie, Dundee DD2 5DA, UK; 2 Cell and Molecular Sciences, James Hutton Institute, Invergowrie, Dundee DD2 5DA, UK; 3 Information and Computational Sciences, James Hutton Institute, Invergowrie, Dundee DD2 5DA, UK

## Abstract

There are currently 151 plants with draft genomes available but levels of functional annotation for putative protein products are low. Therefore, accurate computational predictions are essential to annotate genomes in the first instance, and to provide focus for the more costly and time consuming functional assays that follow. DNA-binding proteins are an important class of proteins that require annotation, but current computational methods are not applicable for genome wide predictions in plant species. Here, we explore the use of species and lineage specific models for the prediction of DNA-binding proteins in plants. We show that a species specific support vector machine model based on *Arabidopsis* sequence data is more accurate (accuracy 81%) than a generic model (74%), and based on this we develop a plant specific model for predicting DNA-binding proteins. We apply this model to the tomato proteome and demonstrate its ability to perform accurate high-throughput prediction of DNA-binding proteins. In doing so, we have annotated 36 currently uncharacterised proteins by assigning a putative DNA-binding function. Our model is publically available and we propose it be used in combination with existing tools to help increase annotation levels of DNA-binding proteins encoded in plant genomes.

## INTRODUCTION

There are currently 151 draft plant genomes available in the NCBI genome database (accessed 09/02/15), collectively representing more than 115 gigabases. However, a genome is only of value if genes can be identified and functions assigned. Assigning functions to the protein products of genes is a complex task, partly as function can be defined on different levels, including cellular location, molecular functions, biological processes (1), as well as post-translational modifications. Using experimental assays to assign function, whilst accurate, is both difficult, costly and time consuming, and currently can not keep pace with the volume of sequenced data (2). Therefore, accurate computational predictions are essential to annotate genomes in the first instance, and to provide focus for functional assays in downstream analysis.

Gene Ontology (GO) terms (1) can be used to assign molecular functions to proteins to assess levels of genome annotation. On average 44% of proteins within the Viridiplantae (Green plants) are annotated with molecular function GO terms, with levels varying from 33% for the moss *Physconitrella patens*, to 56% for the model species *Arabidopsis thaliana*. Economically important crop plants also have low levels of molecular function annotation: *Oryza sativa* (35%), *Solanum tuberosum* (43%), *Zea mays* (45%) and *Solanum lycopersicum* (45%). Therefore, while it is clear that functional annotations on a genomic scale are the key to understanding plants at the molecular level, this is severely limited by the current level of annotation. As a consequence the development of computational protein function prediction methods is a key research area. The first large-scale Critical Assessment of protein Function Annotation (CAFA) experiment featured more than 50 competing algorithms (3), and found that tools which predicted molecular functions provided a greater level of accuracy compared to those which predicted a protein's involvement in a biological process. Overall, the prediction algorithms were less accurate at predicting the function of multi-domain proteins compared to single-domain proteins, which led to the conclusion that algorithms needed to be developed to make predictions for specific molecular functions for multi-domain proteins. One group of proteins which are predominantly multi-domained (4), and are of particular importance in eukaryotic biology, are those that bind DNA.

It is estimated that DNA-binding proteins (DNA-BPs) comprise 6–7% of eukaryotic proteomes (5). They are crucial for many fundamental cellular processes, including replication, transcriptional control, chromatin stability and modification, and epigenetic regulation (6,7). The large number of DNA-binding protein families, and the diverse DNA features that these proteins recognise makes identification of DNA-BPs within proteomes a difficult task (5,8). Prediction of DNA-BPs has been approached using information from protein sequences and/or protein structures. Prediction tools can be divided into three groups, those that (i) use protein sequence information to make DNA-binding predictions at the protein level, (ii) use protein structural information to make DNA-binding predictions at the protein level and (iii) use protein sequence information to make predictions as to which residues interact with DNA, but do not give a prediction of DNA-binding at the protein level. In addition there are tools that use combinations of these approaches. Tools in group (i) are summarised in Table 1, and tools representing the other groups have been reviewed previously (9). Although structure based predictions are more accurate than sequence based ones, the lack of structural information for many proteins makes this method inappropriate for high-throughput annotation. Hence, many existing methods have focused on predictions at the whole protein level using amino acid sequence information, and many of these apply supervised machine learning methods (9–21).

**Table 1.** Summary of previously published methods to predict DNA binding proteins from amino acid sequence

Name	Algorithm	Sequence similarity required?	Training data sets	Availability	Max Seqs
DNAbinder (10)	SVM	None	PDB (146) <= 25% seq id	http://www.imtech.res.in/raghava/dnabinder/	Unlimited
PseAA (11)	SVM	None	PDB (118) < 35% seq id	Not publicly available	Unknown
SVM-SMO (12)	SVM	PSSMs	SwissProt (6653) < 40% seq id	Not publicly available	Unknown
Hybrid Fractal features (13)	SVM	None	PDB (146) [10] <= 25% seq id	Not publicly available	Unknown
mRMR-IFS/ Ensembl learning (14)	SVM	PSSMs	PDB (231) <40%	Not publicly available	Unknown
iDNA-ProtIdis (15)	SVM	None	PDB (525) <25% seq id	http://bioinformatics.hitsz.edu.cn/iDNA-Prot_dis/	50
newDNA-Prot (16)	SVM	PSSMs	PDB (146) [10] and (18)	Fortran code for Windows http://sourceforge.net/projects/newdnaprot/	1
DNA-Prot (17)	Random forest	None	PDB (146) (10)	http://www3.ntu.edu.sg/home/EPNSugan/index_files/dnaprot.htm. Dependant on R - randomForest package but incompatible with R4.6–10	Unknown
iDNA-Prot (18)	Random forest	None	PDB (212) <25% seq id	http://www.jci-bioinfo.cn/iDNA-Prot	50
DBPPred (19)	Random forest + naive Bayes	PSSMs	PDB (390) <25% seq id	Python scripts. Dependent on SPINE-X (54) - unavailable	Unknown
Boosted trees (20)	Decision tree	None	PDB and NDB. 7 data sets (20–35% seq id)	Dependent on Malibu workbench (55) - unavailable	Unknown
DBD-Threader (21)	Threading	Fold similarity	PDB (179) <35% seq id	http://cssb.biology.gatech.edu/skolnick/webservice/DBD-Threader/	1

Supervised machine learning involves the use of algorithms which can learn from validated data sets how to make predictions on new data. Many machine learning methods have been used to predict protein function from amino acids sequence information, but support vector machines (SVMs) have proved in many circumstances to have the best performance (22). SVMs are a supervised learning method used for data classification. Using a set of features which describe the data, the SVM learns to classify training data, where the class value is already known, to create a prediction model. The resulting model can then be applied to data where the class is unknown to predict the values (23). SVMs are specifically suited to the problem of predicting function from sequence as they can be trained on many different types of data, they are suited to noisy data sets, and are less susceptible to overfitting (24). When predicting DNA-BPs it is important to have a high quality data set to train the SVM, and a feature vector which adequately describes the data. One of the simplest and most common features selected to predict protein function is amino acid composition.

A number of models have previously been developed to predict DNA-BPs from amino acid sequence (summarised in Table 1), but most have many limitations that restrict their application to whole genome DNA-BP annotation in plants. We identified five limitations shared by previous models: (i) the use of sequences from mixed prokaryotic, eukaryotic and species data sets for training, (ii) the restriction of training data sets to proteins with solved structures bound to DNA, (iii) reliance upon evolutionary relationships evident in position-specific-scoring matrices (PSSMs), (iv) use of complex models with large numbers of feature vectors giving slow running times, (v) lack of publically available website or software suitable for whole genome predictions.

The development of models based on proteins from a wide range of eukaryotic and prokaryotic species results in generic models, but these often lack the specificity required to annotate lineage specific DNA-BPs. For example, transcription factors are known to be highly specific to each kingdom, with up to 47% of DNA binding transcription factors belonging to lineage-specific families (25). In addition, RNA-binding proteins have been shown to have amino acid binding propensities that are lineage specific (26). Despite such observations, very few studies have been conducted on the use of lineage specific models. One study analysed the potential of using lineage specific models in prokaryotes, but this was limited to a small number of closely related bacteria (27). Hence, there is a need to assess the performance of species/lineage specific prediction models for DNA-BPs.

The use of DNA-BPs for which there is a solved structure of the protein–DNA complex available in the Protein Data Bank (PDB), severely restricts the number of proteins that can be used to effectively train an SVM. This also impacts on the potential to implement a lineage/species specific approach. The use of evolutionary profiles as a feature vector for predictions represents an additional problem when predictions are required for non-model organisms. Many plants have few close relatives with sequenced genomes, meaning that useful PSSMs can not be created, limiting the use of prediction methods that use evolutionary profiles.

The prediction algorithms surveyed in Table 1, show a trend for increasingly complex models based on large numbers of sequence features (e.g. Zhang *et al*. (16) use a 128 feature model and Zou *et al*. (14) use a 153 feature model), using more complex concepts from computer sciences (e.g. chaos game theory (13)). Whilst such models can be shown to have increased accuracy on test data sets, they are primarily focussed on theoretical method development rather than application. This complexity hinders the application of these models to proteome wide predictions, due to their long running time and limitations on the number of input sequences. The final limitation of many of the prediction algorithms is that they are no longer publically available or have software dependencies that are no longer functioning. Of the 12 algorithms surveyed, only DNAbinder (10) could be applied to whole proteome function prediction.

In this work we develop a prediction tool that overcomes these limitations and is applicable to the prediction of DNA-BPs in plant genomes. We have created species and lineage specific models using experimentally validated DNA-BPs, selected using molecular function GO terms. The model only uses a single feature vector, amino acid composition, and this combined with its implementation through the WEKA workbench software (28) means we have created a plant genome annotation tool that can be used by others on genome sequences. For the development of our model we firstly demonstrate that using species specific prediction models, with the dicotyledonous model plant *Arabidopsis* (*Arabidopsis thaliana*) and yeast (*Saccharomyces cerevisiae*), gives more accurate predictions than the generic DNAbinder model (10). Building upon this we created a plant specific model and tested its application to realistic data sets, designed to simulate the relative proportion of DNA-BPs within a plant genome. This allowed us to show that our predictions still perform well when there is not an equal number of DNA-binding and non-DNA-BPs. Finally, we demonstrate the application of the plant model to the prediction of DNA-BPs in the tomato (*Solanum lycopersicum*) genome. Tomato is an economically important crop plant which is used as a model species for fruit development and whose draft genome has been recently sequenced (29). Analysis of GO annotation of the genome reveals that only 43% of proteins have a molecular function assigned, and hence this provided a good system on which to apply our model. We made proteome wide predictions for the tomato genome, and compared the results to current annotations and results from mass spectrometry analysis of chromatin-associated protein fractions. These predictions reveal a large number of tomato proteins (1459) that have possible DNA binding activity and from these, we highlight 36 currently uncharacterised proteins, which we propose to be putative DNA-BPs.

## MATERIALS AND METHODS

### Data sets

**Figure 1.**
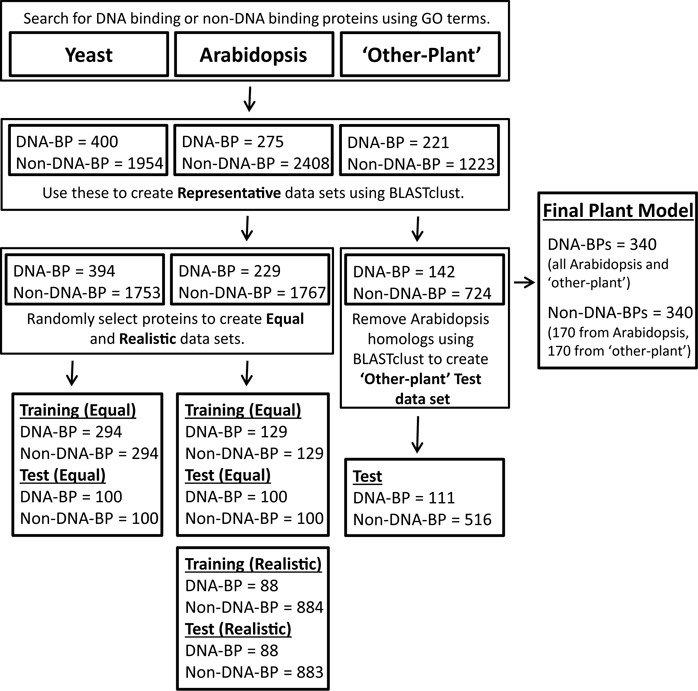
Workflow of data set extraction. Summary of the methods used to obtain protein sequences and create each data set.

Three types of data set were used to create and evaluate our species and lineage specific prediction models. Details of their creation and composition are summarised in Figure 1 and detailed below.

- **Equal data sets:** Full length protein sequences were obtained from the Gene Ontology database (1) using AmiGO (version 1.8) (30), by searching for *Arabidopsis* (*A. thaliana*) (accessed: 07/12/13) and yeast (*S. cerevisiae*) (accessed: 26/01/14) proteins which had a molecular function with an experimental evidence code of IDA (function inferred by direct assay). DNA-BPs were selected using GO terms associated with DNA binding (GO:0003677, GO:0003700, and their child terms). Proteins less than 40 amino acids in length were removed to ensure only full length proteins were present. BLASTclust, from the NCBI-BLAST package (31), was then used to remove homologous proteins (with ≥35% sequence identity over ≥90% sequence length) and a sequence was selected randomly from each cluster to give a representative data set (Supplementary File 1). Final training sets were obtained by randomly selecting 294 DNA-BPs and 294 non-DNA-BPs from our yeast representative data set, and 129 DNA-BPs and 129 non-DNA-BPs from our *Arabidopsis* representative data set. The remaining 100 DNA-BPs for each species were used, along with 100 randomly selected remaining non-DNA-BPs to create data sets for testing. This allowed us to maximise the number of DNA-BPs used to create these models while retaining a suitable number of proteins for testing.
- **Realistic data set:** In reality proteomes comprise many more non-DNA-BPs than DNA-BPs (the estimated ratio is 10:1), and hence a realistic data set for *Arabidopsis* was created to reflect this. The workflow used to identify non-DNA-BPs from *Arabidopsis* gave a total number of 1767 non-homologous proteins. This set was divided in two, giving 884 non-DNA-BPs for the training set, and 883 for the test set. To give a ratio of 10:1 the maximum number of DNA-BPs used was 88 in both the training and testing data sets.
- **‘Other-Plant’ data set:** To determine how well the *Arabidopsis* model performed predicting DNA-BPs from other plant species, a data set which included ‘other-plant’ species was created. Proteins from all Viridiplantae species (excluding *Arabidopsis*) with a DNA-binding molecular function GO term (GO:0003677, GO:0003700, and their child terms) and manually annotated evidence code were extracted using QuickGO (32). A non-DNA-BP data set was extracted in a similar way, except the GO terms selected were not associated with DNA-binding. Proteins with less than 40 amino acids in length were removed. In addition proteins with homologs (with ≥35% sequence identity over ≥90% sequence length) within the data set and to proteins in the *Arabidopsis* Representative data set were removed. This resulted in a final representative ‘other-plant‘ data set consisting of 111 DNA-BPs and 516 non-DNA-BPs (Supplementary File 1). The ‘other-plant’ data set was then used as an independent test set to evaluate the performance of the *Arabidopsis* model.

### Support vector machine (SVM) prediction models

The SVM models developed to predict DNA-BPs from protein sequence information were created using WEKA, a software workbench that integrates a collection of machine learning tools (28). We used the libSVM library (33) with the Radial Basis Function (RBF) kernel (34). The RBF kernel nonlinearly maps data into a higher dimensional space, so it can be used for data in which the relationship between class labels and attributes are not linear, as is the case for the amino acid sequence data used in our model. The RBF kernel has been used in many other SVMs for prediction of protein–DNA interactions (11,14,15) and protein–RNA interactions (35), and hence we selected it to test our models. In each case, we created our models by optimising the cost and γ parameters, using a grid search on the training data.

### Evaluation of SVM prediction models

Five-fold cross validation has been used to evaluate the performance of our models. This involves randomly splitting the data set into five equal groups. Four of these groups are used for training while the remaining group is used for testing. This process is repeated five times so that each group is used once for testing.

Using the numbers of true positives (TP), true negatives (TN), false positives (FP) and false negatives (FN), four statistics were calculated to determine the SVM performance:

- Accuracy = $\frac{{({\rm TP + TN})}}{{({\rm TP + TN + FP + FN)}}}$, measure of the overall number of correct predictions
- Sensitivity = $\frac{{\rm TP}}{{\rm (TP + FN)}}$, measure of correct true positive predictions
- Specificity = $\frac{{\rm TN}}{{\rm (TN + FP)}}$, measure of correct true negative predictions
- Matthews correlation coefficient (MCC) = $\frac{{\rm (TP \times TN) - (FN \times FP)}}{{\sqrt {\rm (TP + FN)(TP + FP)(TN + FN)(TN + FP)} }}$, measure of the quality of the classification.

Our species specific models created with equal data sets were benchmarked against the Alternate DNAbinder model using a threshold of 0.2, the optimal parameter (10). This model was used as the benchmark as it is based on equal sized training and testing sets of full-length sequences.

### SVM plant model and its application to tomato genome annotation

A plant lineage specific SVM model was created using a data set of all 340 plant DNA-BPs and 340 randomly selected non-DNA-BPs from the *Arabidopsis* and ‘other-plant’ representative data sets (170 from *Arabidopsis*, 170 from ‘other-plant’) (Figure 1). This plant lineage model was applied to the tomato (*Solanum lycopersicum*) genome (release ITAG2.4), which is comprised of 34725 proteins. A probability score (ranging from 0.5 to 1.0) was assigned by the model to each predicted protein. This score represents the likelihood of that prediction being correct. For the tomato proteome we used a probability score ≥0.85 to designate putative DNA-BPs. Further details of this model and the data sets can be found in Supplementary Files 2–4.

### Annotation comparisons for tomato genome predictions

The proteins predicted to be putative DNA-BPs were assessed by extracting information from the current tomato genome annotations (29) which included GO terms and assigned Interpro domains, and by assigning Pfam domains in a separate process (36). In this way we assigned proteins to one of three sets: GOA-DB (Gene Ontology Annotation - DNA binding), GOA-Other and GOA-Unknown. Proteins which had evidence of being DNA-binding were designated as GOA-DB and those which had alternative molecular functions were designated as GOA-Other. Proteins that either had no GO term or Interpro domain, or had annotations with insufficient information to assign function were designated as GOA-Unknown.

### Nuclear localisation enrichment analysis of tomato genome predictions

In order to further assess the likelihood of the 1459 predicted proteins having a DNA-binding function, we used WoLF PSORT (37), to predict which proteins were localised to the nucleus. We used all proteins ranked 1 by WoLF PSORT (37) (the highest level of confidence for nuclear localisation) to calculate a nuclear localisation enrichment score. This score was calculated as

\begin{equation*}\frac{{{\rm nNL}}_{{\rm DNA} - {\rm BP}} / {{\rm nDNA} - {\rm BP}}} {{{\rm nNL}_{{\rm T}\;{\rm Prot}}} / {{\rm nTProt}}}\end{equation*}

where:

nNL_DNA-BP_ = number of predicted nuclear localised proteins in 1459 putative DNA-BPs

nDNA-BP = number of putative DNA-BPs = 1459

nNL_TProt_ = number of predicted nuclear localised proteins in tomato proteome

nTProt = number of proteins in tomato proteome = 34725.

### GO enrichment analysis of tomato genome predictions

To determine which Gene Ontology molecular function terms were significantly enriched in our predicted DNA-BPs from the tomato proteome, we used agriGO (38) to conduct singular enrichment analysis (SEA). Any proteins without an associated GO term were removed from the analysis. The GO enrichment analysis was also conducted on a reference set of GO terms in the tomato proteome for comparison. Significant results (*p<0.05*) were determined using Fisher's exact test with false discovery rate correction.

### Experimental validation of DNA-BP predictions in tomato

We initially evaluated the predicted DNA-BP proteins from tomato against current electronic annotations in the Gene Ontology to assess the overlap between known and predicted DNA-BPs. Whilst this was a useful first step, protein function can only be confirmed using experimental assays. However, the major problem for this work was the fact that a high throughput assay for direct DNA-binding in tomato proteins is not feasible. However, the identification of proteins associated with chromatin is possible. Chromatin is a macromolecule comprising proteins, DNAs and RNAs; and within this structure only a subset of proteins will directly bind to DNA. Hence the identification of proteins associated with chromatin is one essential first step in the experimental validation of DNA-binding function of tomato proteins. Whilst identifying a protein as chromatin-associated does not definitively define it as a DNA-BP, it provides a means of prioritising proteins for further experimental investigation. The identification of proteins as chromatin-associated involved three steps; chromatin fractionation, mass spectroscopy and Western blotting.

### Chromatin fractionation

Chromatin-associated and non-chromatin-associated protein samples from tomato tissues were prepared for mass spectrometry analysis using the chromatin fractionation method, as follows:

Tomato leaf tissue was harvested and ground under liquid nitrogen. Ground leaf tissue was suspended in 10 ml ice cold buffer (10 mM PIPES, 10 mM KCl, 1.5 mM MgCl_2_, 340 mM sucrose, 10% glycerol, 0.5% Triton X-100 and 1X SIGMAFAST protease inhibitor cocktail (Sigma), pH 6.8) and filtered through Miracloth (Calbiochem). A 6 ml aliquot of this whole cell extract was taken and centrifuged at 3000g for 10 min at 4°C. 1 ml of TCA-A (10 ml acetone, 2 ml TCA (20% w/v in H_2_O), 8 μl β-mercaptoethanol) was used to resuspend the pellet, containing chromatin bound proteins. After brief vortexing, these samples were stored at −20°C for 1 h. Samples were centrifuged at 16000g for 30 min at 4°C and the resulting pellets were washed three times with a/β-me solution (8 μl β-mercaptoethanol in 10 ml acetone) before resuspending in 100 μl sodium dodecyl sulphate loading buffer.

### Mass spectrometry

Three chromatin fractionation replicates were processed for mass spectrometry as follows:

Non-chromatin associated and chromatin associated protein samples were loaded onto 4–20% precast polyacrylamide gels (BioRad) and the samples run $\frac{2}{3}$ of the gel. Each lane was cut into six equally sized pieces, with each section then cut into 1 mm square cubes. Gel pieces were washed with 100 mM NH_4_HCO_3_ and acetonitrile followed by reduction with 10 mM DTT and alkylation with 55 mM iodoacetamide. Proteins were subject to in-gel digestion with sequencing grade trypsin (Roche) at 37°C overnight, generating peptides. A C18 (POROS R2, Applied Biosystems) column, which had been activated with 50% acetonitrile, 0.1% trifluro acetic acid (TFA) and washed with 0.1% TFA, was used to clean the peptides. Peptides were loaded onto the column and washed with 0.1% TFA. Peptides bound to the column were eluted using 2 × 40 μl of 50% aectonitrile, 0.1% TFA. Peptide samples were dried down to approximately 10 μl using a vacuum centrifuge before adjusting to a final volume of 30 μl using 0.1% TFA.

Peptide samples were run on an LTQ Orbitrap Velos Pro (Thermo Scientific) mass spectrometer, on a 95 min run. The MS resolution was set to (FTMS) 60 000. MS/MS was done on the top 16 ions, using Collision Induced Dissociation (energy 35) on a minimum signal of 5000, with an isolation window of 2 ppm. Unassigned and 1+ ions were rejected. Dynamic exclusion was used with a repeat count of 1, repeat duration of 30 s and exclusion duration of 45 s.

Protein identification and quantification was done using MaxQuant software version 1.4.1.2 (39). Raw data files were searched against the predicted proteins of the *S. lycopersicum* genome ITAG2.3 release. The resulting data were then filtered to obtain all chromatin associated proteins which were found in at least one replicate.

### Western blotting

To assess the levels of nuclear and non-nuclear proteins in our mass spectrometry samples, we carried out Western blots using specific antibodies. Tomato leaf tissue chromatin fractionation samples, which were submitted for mass spectrometry analysis, were run on Biorad TGX gels before being transferred to PVDF membranes with the Biorad Trans Blot Turbo System. Blots were blocked for 30 min with 5% milk in TBS-T (0.1% Tween 20) before being probed with Calnexin (endoplasmic reticulum protein), UGPase (cytoplasmic protein) or Histone H3 primary antibody. The blots were washed three times with TBS-T for 10 min before addition of the corresponding secondary antibodies, and leaving for 2 h. Blots were washed with TBS-T three times for 10 min before incubation with SuperSignal West Femto Maximum Sensitivity substrate (Thermo Scientific) and imaging with a Syngene G:Box TX4 Imager. After imaging Coomassie brilliant blue (Cbb) stain was used on each membrane to visualise total protein levels and the level of RuBisCO in each sample.

## RESULTS AND DISCUSSION

In this work we have shown that species-specific models for the prediction of DNA-binding proteins outperform a generic model based on proteins from multiple species (DNAbinder (10)). We have used this result to create a plant specific model, and applied it to the prediction of DNA-BPs in the tomato genome. In doing so we have assigned putative DNA-binding function to 36 currently uncharacterised tomato proteins. This is the first time species-specific SVM models have been shown to perform better than generic models for DNA-BP function prediction, and this has led to a high throughput DNA-BP prediction tool suitable for plant genomes.

### Species-specific models perform better than a generic model

We hypothesised that a species-specific DNA-BP prediction model would give more accurate predictions for proteins from that species, than a generic model. To test this we created *Arabidopsis* and yeast models using equal numbers of DNA-binding and non-DNA-binding proteins (Figure 1). We then compared these against the generic DNAbinder model, which is trained on a mixed data set of eukaryotic and prokaryotic DNA-BPs (10). The 5-fold cross validation of these models showed that our *Arabidopsis* and yeast specific models have an increased accuracy and MCC compared to DNAbinder (Table 2). To substantiate this finding we evaluated our yeast model on our *Arabidopsis* data set and vice versa (Table 2). This confirmed that prediction accuracy was reduced when non-species specific models were used.

**Table 2.** Evaluation statistics of species specific libSVM models and comparison with DNAbinder model using equal data sets

libSVM model	Test data set	Accuracy	Sensitivity	Specificity	MCC
*Arabidopsis*	*Arabidopsis*	0.81	0.77	0.85	0.62
Yeast	Yeast	0.76	0.81	0.71	0.52
*Arabidopsis*	Yeast	0.74	0.66	0.81	0.48
DNAbinder (10)	*Arabidopsis*	0.74	0.75	0.72	0.47
Yeast	*Arabidopsis*	0.67	0.53	0.81	0.35
DNAbinder (10)	Yeast	0.65	0.84	0.47	0.33

Results are shown in order of decreasing Matthews Correlation Coefficient (MCC).

These results raise the question of why species-specific models give more accurate predictions. It is known that a number of DNA-binding protein families, such as histones and core transcription factors (40,41), are conserved across diverse lineages. However, there are also families of DNA-BPs and transcriptional regulators which are highly specific to eukaryotic lineages and participate in lineage specific processes (42). Most relevant to the current results are the many plant specific families of DNA binding transcription factors (TFs) (43). *Arabidopsis*, for example has a large repertoire of TFs, 45% of which are lineage specific (25). If training sets of representative DNA-BPs are extracted from diverse species then the models based on them may lack sequence information from species-specific proteins. This is an important factor especially relevant for DNA-BP prediction in non-model species, as many existing prediction methods rely upon sequence similarity for some aspect of the prediction (Table 1).

Another factor that will influence the increased accuracy of the species-specific models, is the fact that DNAbinder is only trained on DNA-BPs that have solved 3-D structures of the protein in complex with DNA. The SQUAMOSA promoter binding (SPB) protein and B3 are both plant specific transcription factors (included in our *Arabidopsis* DNA-BP data set) that do not have a crystal structure available in complex with DNA. Hence these plant-specific DNA-BPs are excluded from models based on protein structures; which includes 11 of the 12 existing models summarised in Table 1. Our models are trained on protein sequences with GO annotations, and are not restricted to proteins with DNA bound structures, ensuring that many important lineage specific DNA-binding families are included within the model, thus contributing to their increased accuracy.

### Performance of *Arabidopsis* SVM model on the realistic data sets

The *Arabidopsis* model based on a realistic data set achieved an accuracy of 91% and a MCC of 0.51, whilst retaining a high sensitivity (Table 3). This showed that the effectiveness of using species-specific models is retained when making predictions for realistic data sets. This is an important result if the models are to be used on a genomic scale, where DNA-BPs will form only a small percentage of the total protein content.

**Table 3.** Evaluation statistics of the realistic *Arabidopsis* model tested on realistic *Arabidopsis* and ‘other-plant’ data sets

libSVM model	Test data set	Accuracy	Sensitivity	Specificity	MCC
*Arabidopsis*	*Arabidopsis*	0.91	0.61	0.94	0.51
*Arabidopsis*	‘Other-Plant’	0.80	0.27	0.91	0.2

Results are shown in order of decreasing Matthews Correlation Coefficient (MCC).

### Performance of *Arabidopsis* SVM model on the ‘other-plant’ data set

**Figure 2.**
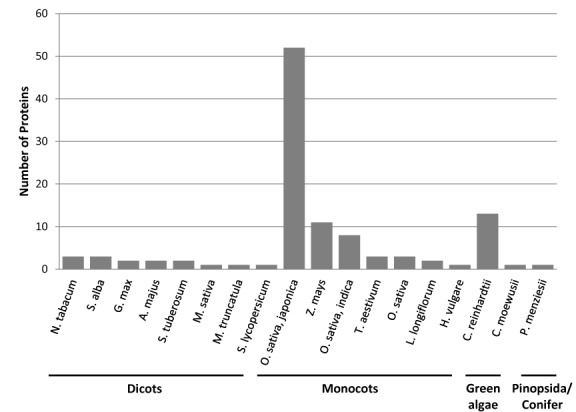
DNA-BPs in Other Plant data set. Bar chart showing the species distribution of the DNA-BPs in the ‘Other-plant’ data set.

In total 1322 DNA-BPs were extracted from all available Viridiplantae genomes, but only 8.4% were from species other than *Arabidopsis* (Figure 2). A large proportion of the additional proteins were from rice (*Oryza sativa*), which has a high level of manual annotations, most likely reflective of its status as a model organism for cereals (Figure 2). These data provide further evidence that there is a need for high throughput methods for protein annotation in plants.

When evaluated against this ‘other-plant’ data set, our *Arabidopsis* model achieved reasonable accuracy (80%) but a very low sensitivity (0.27) (Table 3); suggesting that the model is not suitable for predictions of DNA-BPs from evolutionary diverse plant species. One possible explanation for this is that there are a significant number of species-specific DNA-binding proteins that are not included within our *Arabidopsis* training data. This could be reflective of the high proportion of monocots in the ‘other-plant’ data set, when the model is based on the dicot *Arabidopsis*. Whilst there are TFs which are shared between monocots and dicots, there are also proteins which are specific to each (44). However, BLASTP analysis of our ‘other-plant’ data set against the *Arabidopsis* proteome (TAIR10 release) revealed that all proteins had a close homolog (e-value<10^−3^) (Supplementary File 5.1). This suggests that the *Arabidopsis* homologs were not in our initial representative *Arabidopsis* data set, possibly due to the lack of an experimentally validated DNA-binding function annotated in the Gene Ontology. Therefore, to ensure that these proteins were represented in our final model we combined our data sets of all the DNA-BPs proteins used from *Arabidopsis* and the ‘other-plant’ data set (340 DNA-BPs) with an equal number of non-DNA-BPs. This data set was used to create a final plant model, suitable for making predictions across diverse plant genomes.

### Prediction of DNA-BPs from tomato using a plant model

The predictions made for the tomato proteome (*S. lycopersicum*) (29) using the plant model resulted in 1459 proteins being designated as having putative DNA-binding function (Supplementary File 5.2). These predictions were completed in ∼20 s using the plant SVM in WEKA (28) (run on a laptop with an Intel core i5–3360M @ 2.80GHz processor with 16.0GB RAM). This short execution time shows that the method is suitable for predicting DNA-BP function on a genomic scale. These 1459 proteins predicted to bind DNA were assigned to one of three annotation sets (GOA-DB, GOA-Other, GOA-Unknown). In total 1006 had a function assigned (GOA-DB and GOA-Other), and of these 68.8% were annotated as putative DNA-BPs. Many of the proteins annotated as GOA-Other, had RNA-binding functions or were zinc finger domains putatively involved in protein–protein interactions. Our plant model is based upon amino acid composition, and hence it was to be expected that proteins’ sharing similar sequence characteristics to DNA-BPs were miss-annotated. Like DNA, RNA is a negatively charged and therefore interacting proteins prominently feature positively charged residues within their binding sites (45,46). Similarly, many zinc finger proteins bind DNA whereas others engage exclusively with proteins or RNA molecules (47), leaving it difficult to define binding preference on amino acid composition alone. A further issue with the GO annotations is that many proteins have multiple domains with different functions (4), and as a result one protein may have several GO terms assigned. Therefore, it is still possible that proteins we have designated as GOA-Other may have as yet unassigned DNA binding functions.

**Figure 3.**
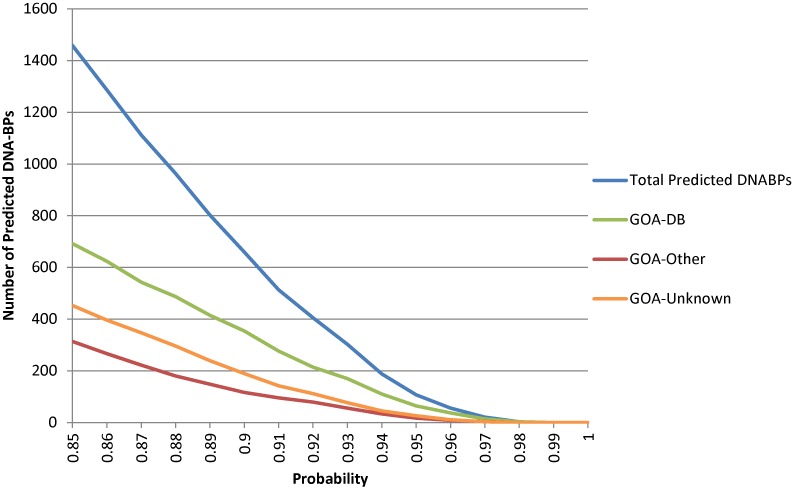
Probability distribution of predicted DNA-BPs from tomato. Line graph showing the relationship between increasing probability and the number of predicted DNABPs (total, GOA-DB, GOA-Other and GOA-Unknown) greater than or equal to the corresponding probability.

Using our final plant model we predicted 1459 DNA-BPs in the tomato proteome using a threshold of ≥0.85. This threshold was chosen based on the number of DNA-BPs predicted. Early estimates suggested that DNA-BPs make up 6–7% of a proteome (5), and for tomato, with a proteome of 34725 proteins, this means that we would expect to see approximately 2084 proteins with DNA-binding function. We made predictions for the tomato proteome with different thresholds, to see which gave a number of putative DNA-BPs close to the expected number (Figure 3). A 0.90 threshold gave a predicted data set of 659 (lower than expected) and at 0.80 the predicted set is 2438 (higher than expected). The probability of 0.85 was used, because it gave a data set size in the region of, but smaller than, the expected. The smaller size was justified as it made manual annotation mappings (which was necessary for some parts of the analysis) possible.

It is also possible for users of our model to test different probability thresholds themselves, to create larger or smaller predicted protein data sets. Figure 3 shows the relationship between the number of predicted DNA-BPs at different probability thresholds, along with the corresponding ratio of GOA-DB, GOA-Other and GOA-Unknown. This shows that as the probability score increases so does the number of GOA-DB compared to GOA-Other. At a probability of ≥0.85 (1459 predicted DNA-BPs) there are 692 GOA-DB (47.4%) compared to 314 GOA-Other (21.5%). When this threshold is increased to ≥0.9 (659 predicted DNA-BPs) there are 354 GOA-DB (53.7%) compared to 116 GOA-Other (17.6%). This ratio of GOA-DB to GOA-Other increases further at a probability ≥0.95. This relationship gives users the option to select a probability threshold that reflects the accuracy and sensitivity of the predictions they wish to make.

**Figure 4.**
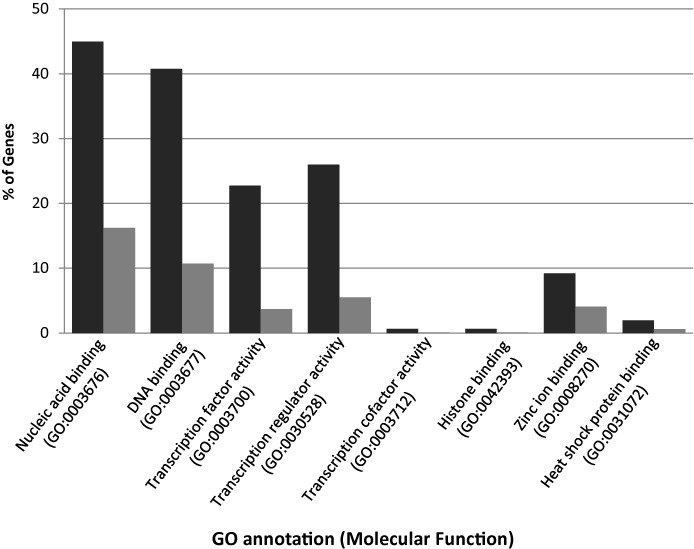
Gene Ontology (GO) term enrichment analysis. Histogram showing the percentage of genes which have a GO term relating to a molecular function which is significantly enriched in our predicted tomato DNA binding proteins (black) compared to the reference tomato proteome (grey).

To further validate the predicted data set, we calculated nuclear localisation (NL) and GO term enrichment scores. The NL enrichment score showed that the 1459 predicted DNA-BPs were 2.9 fold enriched for predicted nuclear localised proteins, compared to the tomato proteome as a whole. This provided further evidence that the plant model identified potential DNA-BPs in tomato. The GO term enrichment analysis, using agriGO (38), was conducted on the 1259 predicted DNA-BPs which had an associated GO term (Figure 4; Supplementary File 5.3). From this analysis it is apparent that our predictions significantly enrich for DNA binding and associated transcriptional molecular processes compared to the tomato proteome. The predicted protein set is enriched for three high level molecular function terms (nucleic acid binding, DNA binding and sequence-specific DNA binding transcription factor activity) as expected, and four additional terms (Figure 4). Three are child terms of protein-binding (transcription cofactor activity, histone binding and heat shock protein binding) and one is a child term of ion-binding. This reflects the fact that proteins binding to DNA often comprise large complexes that include other proteins and molecules. For example transcription factors bind DNA in combination with co-factors (48,49), and zinc finger proteins are one of the largest families of transcription factors in plants where DNA-binding is coordinated by zinc ions (50). Histones are proteins that occur as large protein complexes that fold DNA into structural units called nucleosomes (41). Heat shock proteins are produced during stress responses and many act as chaperones involved in protein folding events (51). In plants, heat shock transcription factors regulate gene expression in response to environmental stress (51,52). Hence, whilst we predict proteins with molecular functions other than DNA-binding, many are related to transcriptional processes where DNA or DNA packaging and modification is involved.

65% of 34725 tomato proteins do not have an annotated molecular function GO term, leaving them as functionally uncharacterised proteins. From the 1459 predicted DNA-BPs in tomato, we have identified 28 proteins with at least one source of evidence for DNA-binding function that are currently annotated as uncharacterised proteins in UniProt Knowledgebase (UniProtKB) (53) (Supplementary File 5.4).

**Figure 5.**
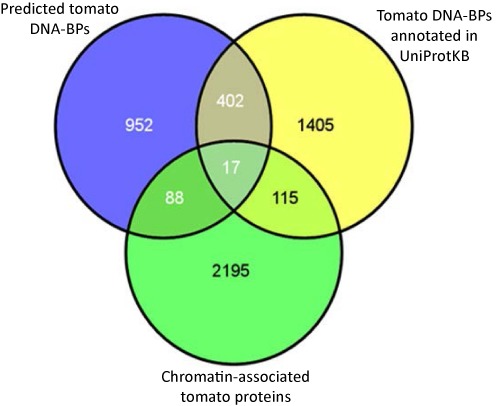
Comparison of predicted tomato DNA-BPs to an existing data set and an experimental data set. Venn diagram showing the number of predicted DNA-BPs from tomato (probability ≥0.85) which are also annotated as DNA binding in UniProtKB (419 proteins) and found in our experimental data set from mass spectrometry analysis of tomato chromatin protein fractions (105 proteins).

The tomato proteome annotation file initially available for our work (ITAG2.4) was generated on 23/02/14, and may have since been superseded by annotations in UniProtKB. Hence, we also compared our 1459 predicted tomato DNA-BPs to 1939 tomato proteins which have a GO term for DNA binding (GO:0003677, GO:0003700) in UniProtKB (accessed 22/09/14) (Figure 5). This shows that only 419 of the predicted DNA-BPs were also found in this UniProtKB data set. The low level of overlap prompted us to further investigate the annotations presented in UniProtKB. In doing so we found that of the 1939 tomato UniProtKB proteins, only 36 are reviewed proteins (i.e. have been reviewed and evidence interpreted by an annotator). The majority of these reviewed protein annotations described multiple members of the same family (i.e. 15 of the 36 reviewed proteins are annotated histones). The remaining un-reviewed proteins (1903 proteins) have annotations that are inferred from electronic annotation, which is an automatically assigned evidence code. This evidence code results from sequence similarity searches and keyword mappings, and hence could be far from robust. This raises the issue that the UniProtKB data set is very inclusive, and potentially comprises large families of proteins that are not DNA-binding. In addition, some of the UniProtKB proteins that do not overlap with our predictions have different annotations from the ITAG2.4 release of the tomato genome. For example, the protein Solyc01g100240.2.1 has a molecular function GO term for calmodulin binding (GO:0005516) as well as a calmodulin binding protein-like domain from Interpro (IPR12416) assigned in the ITAG2.4 release. However, in UniProtKB it is annotated with sequence-specific DNA binding (GO:0043565) and sequence-specific DNA binding transcription factor activity (GO:0003700) GO terms, despite no change in the evidence code or reviewed status. The annotation process and anomalies similar to the one described here, partly explain the relative low level of overlap observed in Figure 5. The low overlap is also partly explained by the final plant model predicting false positives and negatives, as is expected with any machine learning algorithm.

Evaluating our predictions against current electronic annotations is useful as a first step, but ultimately functions can only be confirmed using experimental assays. The lack of a high throughput assay, suited to validate DNA-BPs in tomato led us to use mass spectrometry to identify chromatin-associated proteins from tomato leaf tissue samples, as an initial experimental validation step. The chromatin fractionation and subsequent mass spectrometry led to a data set of 2415 proteins proposed to associate with chromatin. We compared the overlap of proteins in this chromatin-binding data set with the 1459 predicted DNA-BPs and the 1939 DNA-BPs annotated in UniProtKB (Figure 5). From the 952 proteins that have no DNA-binding annotation in UniProtKB, 88 have been shown to have an association with chromatin (Figure 5). Eight of these 88 proteins are currently annotated as uncharacterised in UniProtKB (Supplementary File 5.4), hence we propose they should be annotated as chromatin associated and putative DNA binding proteins.

The overlap between the chromatin-associated proteins and the predicted DNA-BPs is small, and there are a number of reasons for this. The chromatin fractionation assay is not a conclusive approach for experimentally identifying DNA-BPs. Chromatin-associated proteins will include not only DNA-binding proteins but proteins with other functions, many of which will be protein–protein binding. In addition the chromatin fractionation assay indicates potential chromatin-associated proteins by selecting those that are significantly enriched at a certain threshold (Supplementary File 6). Hence, dependent upon the threshold, non-chromatin-associated proteins may also be included. However, to further validate the 2415 proteins identified as chromatin-associated in our assay, we carried out Western blots using the protein samples submitted for mass spectrometry (Supplementary File 6) to see if this set was enriched or depleted in specific proteins. This showed there was a depleted amount of the non-nuclear proteins calnexin, which is found in the endoplasmic reticulum, the cytosolic protein UGPase, and RuBisCO. Importantly, we also see that there is an enrichment of histone H3, one key structural components of chromatin, which interacts with DNA. This suggests that we are enriching specifically for chromatin-associated proteins in our assay (Supplementary File 6).

A further point to highlight is that, DNA-binding is a highly dynamic process. Many different DNA-BPs are involved in the regulation of gene expression, and expression of these proteins may vary in response to different times of the day, different environmental conditions and in different plant tissues. Our chromatin fractionation assay was conducted using a single plant tissue type and at a single time point, hence we know that we are only detecting a small proportion of potential DNA-BPs. We propose that researchers looking to select a reduced number of putative DNA-BPs from the list of 1459 would begin with some of the 88 proteins that overlap with chromatin-associated proteins, but lack UniProtKB annotation.

In this work, we have developed a lineage specific model that allows accurate prediction of DNA-BPs in plant proteomes. This model overcomes the limitations of previous annotation methods, is publically available and capable of high-throughput predictions on a genomic scale. This represents a significant advance in prediction tool development, which will contribute to the annotation of plant genomes in the future. We demonstrated the high throughput capabilities of our plant specific model by making DNA-BP predictions for the whole tomato proteome. This identified a significant percentage of putative DNA-BPs for further analysis and specifically provided predicted DNA-BP annotations for 36 proteins, previously of unknown function. We anticipate that this lineage specific model will form an important complementary genome annotation tool, and suggest that the development of analogous models in other lineages will improve DNA-BP predictions in other systems.

## Supplementary Material

SUPPLEMENTARY DATA
