# Supplementary material for: DNA-binding protein prediction using plant specific support vector machines: validation and application of a new genome annotation tool
Source: Nucleic Acids Res. 2015 Aug 24;43(22):e158. doi: 10.1093/nar/gkv805 (PMC4678848; doi:10.1093/nar/gkv805)
Supplement: SUPPLEMENTARY DATA [file supp_43_22_e158__index.html]

DNA-binding protein prediction using plant specific support vector machines: validation and application of a new genome annotation tool — SUPPLEMENTARY DATA 

# DNA-binding protein prediction using plant specific support vector machines: validation and application of a new genome annotation tool

## SUPPLEMENTARY DATA

- SUPPLEMENTARY DATA
- SUPPLEMENTARY DATA
- SUPPLEMENTARY DATA
- SUPPLEMENTARY DATA
- SUPPLEMENTARY DATA
- SUPPLEMENTARY DATA
- SUPPLEMENTARY DATA
