# Supplementary material for: DNA-binding protein prediction using plant specific support vector machines: validation and application of a new genome annotation tool
Source: Nucleic Acids Res. 2015 Aug 24;43(22):e158. doi: 10.1093/nar/gkv805 (PMC4678848; doi:10.1093/nar/gkv805)
Supplement: SUPPLEMENTARY DATA [file supp_gkv805_nar-01019-met-n-2015-File007.docx]

**Description of Supplementary Files**

**Supplementary File 1**: Multi-fasta file containing the amino acid sequences for the following representative datasets: 394 yeast DNA-BPs, 1753 yeast non-DNA-BPs, 229 *Arabidopsis* DNA-BPs, 1767 *Arabidopsis* non-DNA-BPs, 111 DNA-BPs in our other plant dataset and 516 non-DNA-BPs in our other plant dataset.

**Supplementary File 2**: Perl script used for calculating the percentage amino acid composition of the input protein sequences and converting to .arff format for input into WEKA.

**Supplementary File 3**: File for our plant lineage specific prediction model.

**Supplementary File 4**: Instructions for use of plant specific prediction model in WEKA.

**Supplementary File 5**: Excel file containing tables for the following information. **5.1**: BLAST results for proteins in our other plant dataset against the *Arabidopsis thaliana* proteome. Top hits with the corresponding e-values are shown. **5.2**: 1459 tomato proteins predicted to be DNA binding (probability score ≥0.85). File contains the Solyc id for each protein along with the corresponding functional description and any functional annotations from Gene Ontology, Interpro and the presence of a DNA binding domain predicted by Pfam. **5.3**: All GO terms found in enrichment analysis for our predicted DNA-binding proteins from tomato. The number of times each term appears in the input and reference (tomato proteome) is shown along with the p-value, which was used to select significant enrichment. **5.4**: Uncharacterised tomato proteins which are predicted to be DNA binding as well as having another source of evidence for DNA binding function.

**Supplementary File 6**: Western blot showing depletion of non-nuclear proteins and enrichment of histone H3 in non-chromatin and chromatin associated protein samples taken from tomato leaf tissue. Each of the three replicates used for mass spectrometry were probed with antibodies for calnexin (a protein found in the endoplasmic reticulum), UGPase (a cytosolic protein) and histone H3 (which is associated with DNA). Coomassie brilliant blue (Cbb) stain was used to show a depletion of RuBisCO in chromatin associated protein samples compared to non-chromatin associated protein samples.
