# Supplementary material for: DNA-binding protein prediction using plant specific support vector machines: validation and application of a new genome annotation tool
Source: Nucleic Acids Res. 2015 Aug 24;43(22):e158. doi: 10.1093/nar/gkv805 (PMC4678848; doi:10.1093/nar/gkv805)
Supplement: SUPPLEMENTARY DATA [file supp_gkv805_nar-01019-met-n-2015-File011.pdf]

Supplementary File 4: Instructions for use of our plant specific prediction model in WEKA.

1. Download and install the latest developer version of WEKA for your operating system (http://www.cs.waikato.ac.nz/ml/weka/downloading.html).
2. Using the Perl script provided (Additional data file 6) convert your fasta file of plant amino acid sequences to be predicted to .arff format for input into WEKA.
3. Open the WEKA explorer and open the .arff file created in step 2.
4. Select the classify tab and check “supplied test set” under the test options. Set this as your input .arff file by clicking “set…” -> “open file…” -> select your file -> close.
5. Click on “more options…” and click on “choose” next to output predictions to select the format you would like your output predictions to appear in.
6. Right click on the white result list box and select load model.
7. Open the plant lineage specific model (Additional data file 5).
8. Right click on the model in the results list and select “Re-evaluate model on current test set”. This will run the prediction model on your input dataset.
9. Right click on the model in the results list for the option to save the results buffer containing which proteins are predicted as being DNA binding.
