# Supplementary figures and images for: DNA-binding protein prediction using plant specific support vector machines: validation and application of a new genome annotation tool
Source: Nucleic Acids Res. 2015 Aug 24;43(22):e158. doi: 10.1093/nar/gkv805 (PMC4678848; doi:10.1093/nar/gkv805)

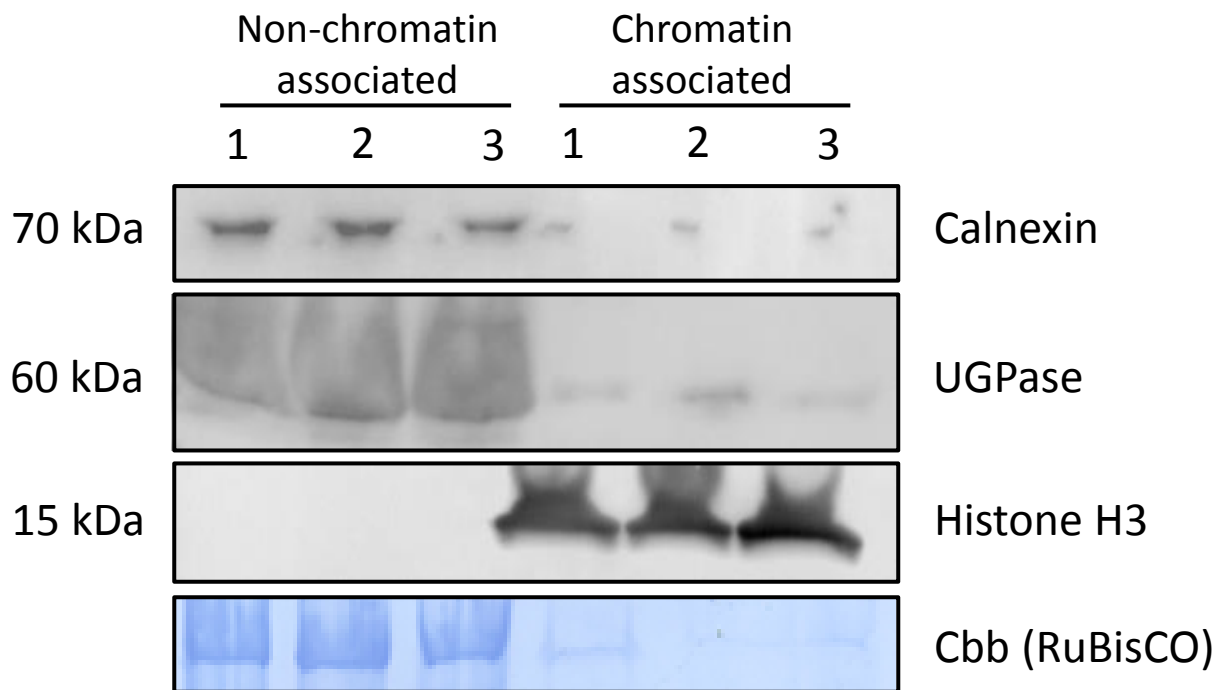

Supplement: SUPPLEMENTARY DATA [file supp_gkv805_nar-01019-met-n-2015-File013.pdf]
